# Supplementary material for: Machine learning reveals genes impacting oxidative stress resistance across yeasts
Source: Nat Commun. 2025 Jul 1;16:5866. doi: 10.1038/s41467-025-60189-3 (PMC12215403; doi:10.1038/s41467-025-60189-3)
Supplement: Supplementary file 1 — Supplementary Information [file 41467_2025_60189_MOESM1_ESM.pdf]

Supplementary Figures

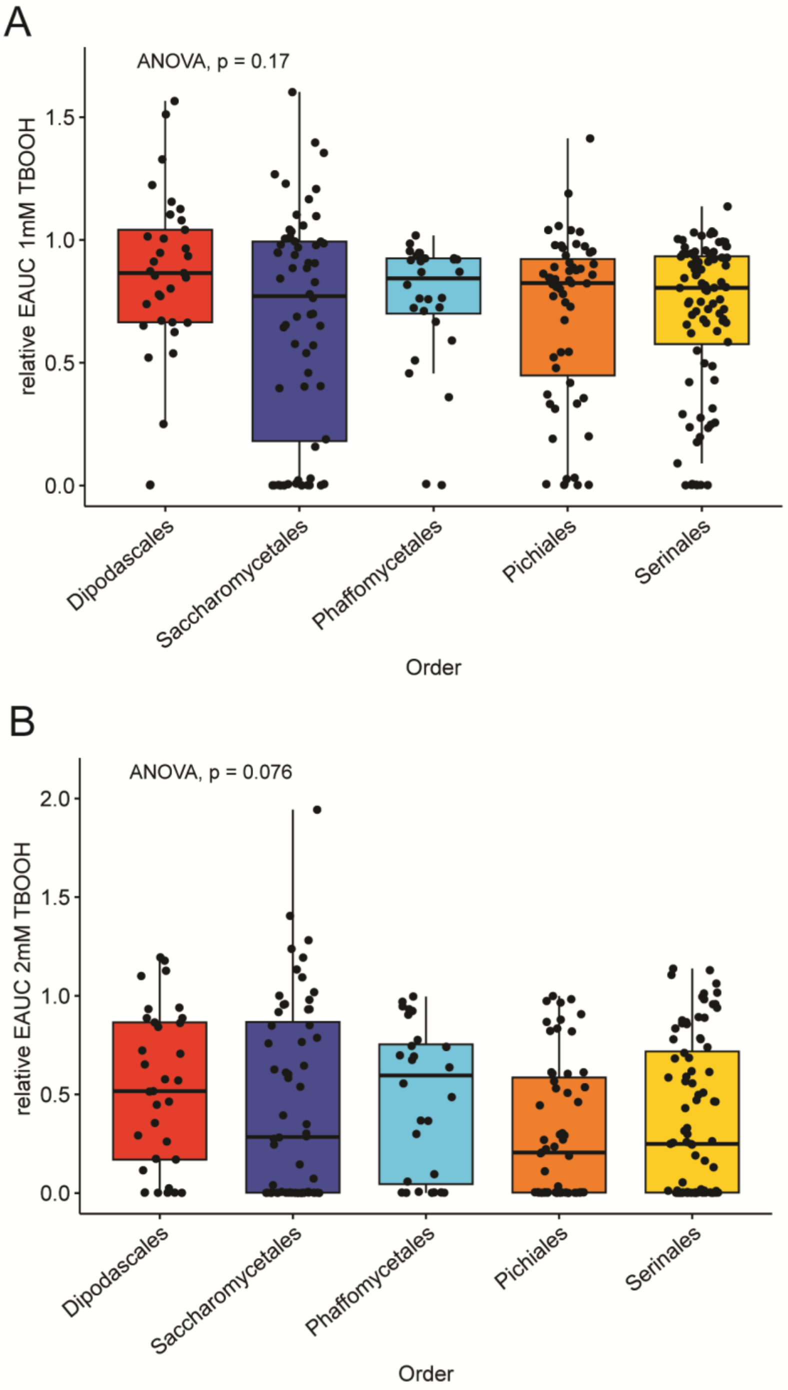

Supplementary Figure 1. The resistance to ROS does not vary significantly among the largest orders. The relative empirical area under the curve (EAUC) for the yeast orders with at least ten species are compared in (A) 1 mM TBOOH and (B) 2 mM TBOOH. The boxes represent the interquartile range of the data and the line in the box represents the median for each order. ANOVAs were used to determine that the orders did not differ significantly.

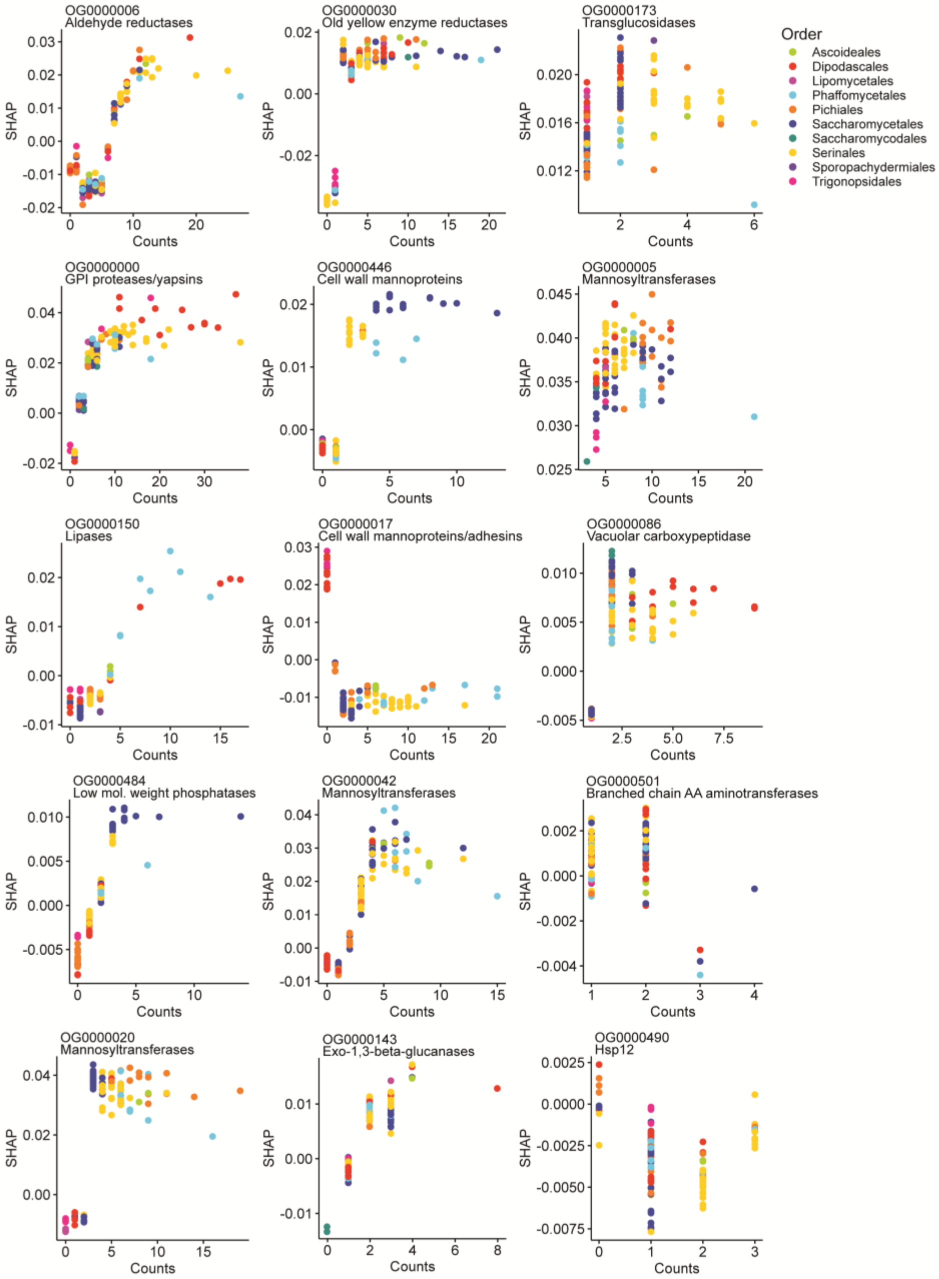

Supplementary Figure 2. The influence of each orthogroup (OG) on the predictions of each species. The association between the number of genes in each OG and the SHAP values for the fifteen most important OGs, as ranked by the machine learning model. Each dot represents a single species, and the color corresponds to the order. Some OGs, such as the *OYE* gene family (OG0000030) had a clear trend wherein species with three or more genes had positive SHAP values. Other gene families, such as the mannosyltransferase-encoding family (OG0000005), had an overall positive trend, but there was not an apparent threshold.

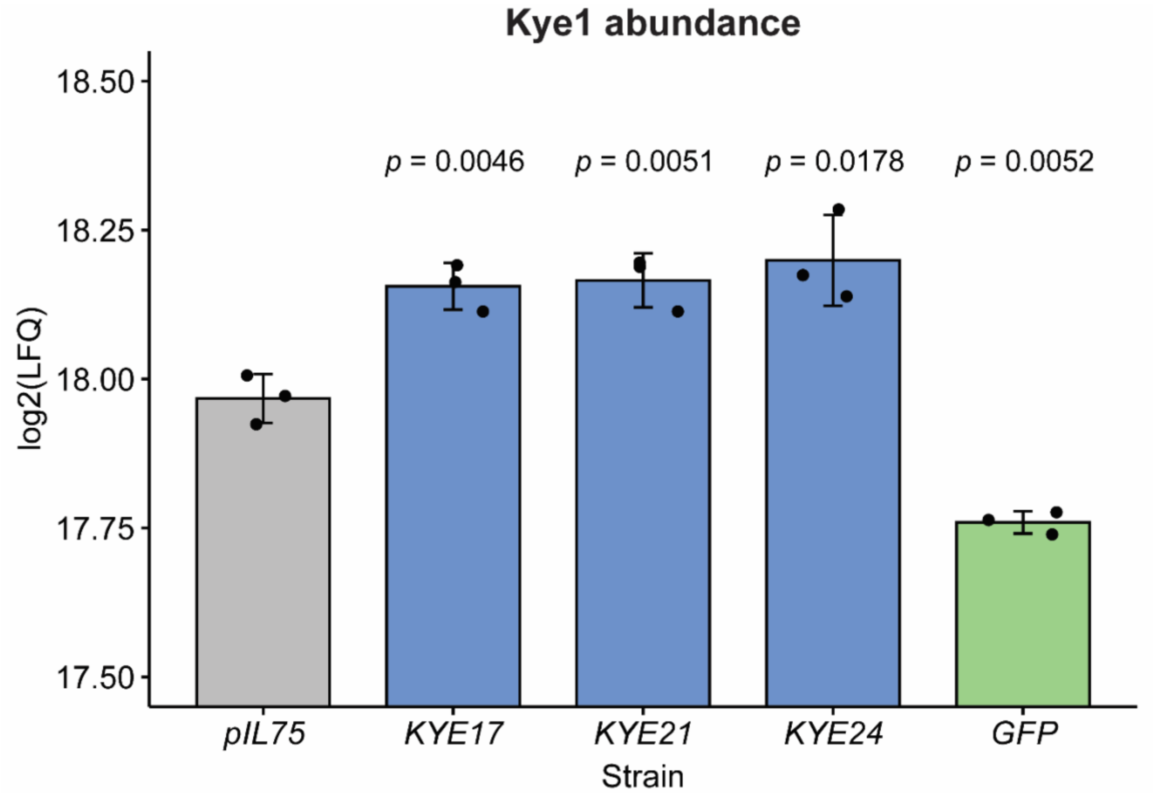

Supplementary Figure 3. The abundance of Kye1 increases in overexpression strains. The three strains of *K. lactis* with an additional episomal copy of *KYE1* all had significantly higher abundance of this enzyme compared to the empty vector (*pIL75).* The height of the bar represents the mean of the log_2-transformed label-free quantification (LFQ) of Kye1 for each strain, and the error bars represent the standard deviation. Each biological replicate (n=3) is shown as an individual dot, and the p-values are based on two-sided t-tests compared to the empty vector control strain.

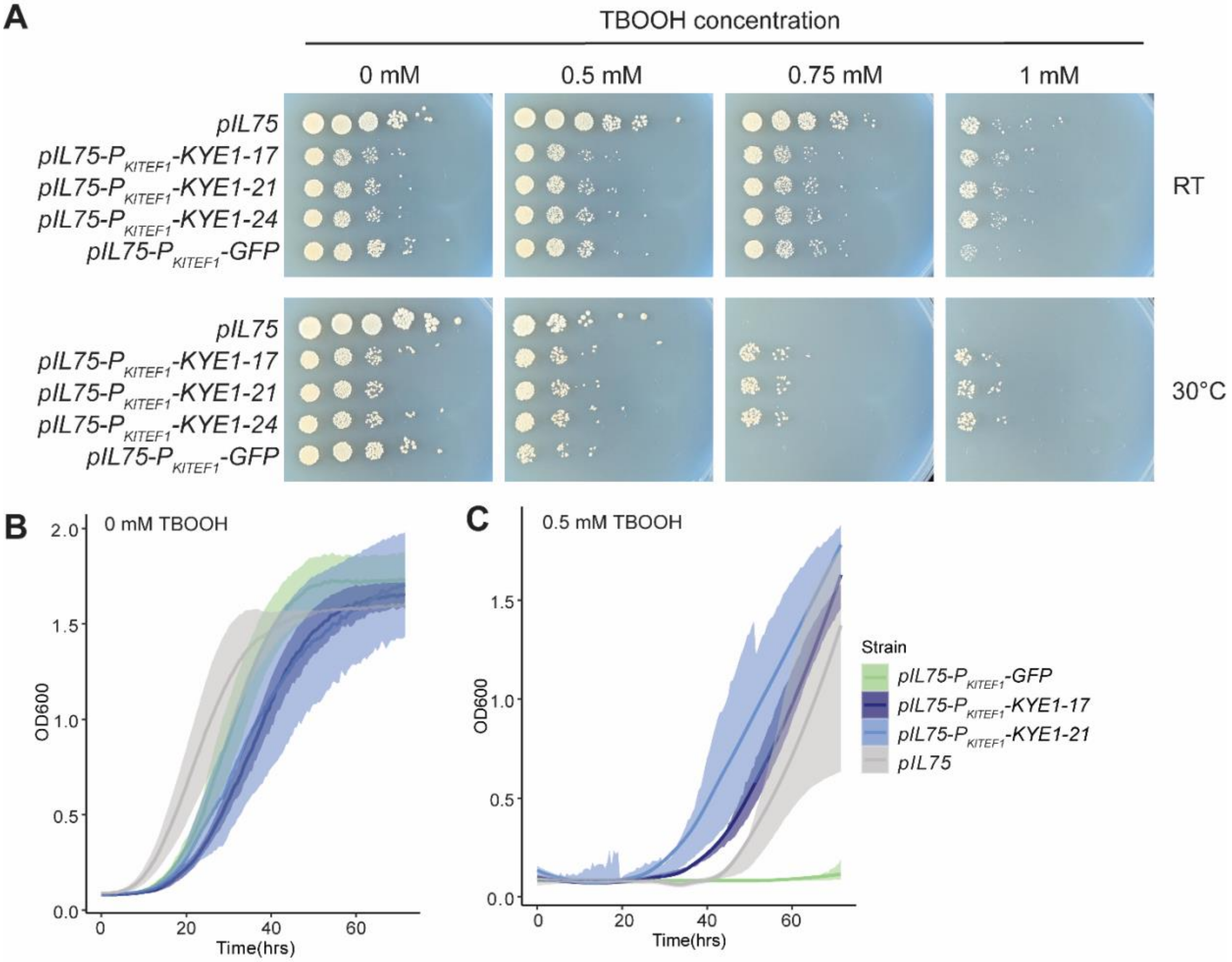

Supplementary Figure 4. *KYE1* overexpression promotes growth in the presence of ROS stress in *Kluyveromyces lactis*. (A) A spot assay showing growth of the *K. lactis* strains transformed with an empty vector (*pIL75*), overexpressing the *KYE1* reductase (*pIL75-pKlTEF1-KYE1*), or overexpressing *GFP* (*pIL75-pKlTEF1-GFP*) to control for the effect of protein overexpression. Plates were supplemented with varying concentrations of TBOOH as indicated. Plates were incubated at two temperatures: room temperature (RT or 22°C) to reflect our screening conditions or 30°C, which is the normal laboratory growth temperature for *K. lactis*. Plates were imaged after four days of growth. Spot assays shown are representative of three replicates. (B, C) Quantitative growth experiments were conducted in liquid medium in 96-well plate format. Growth curves in (B) medium without TBOOH and (C) supplemented with 0.5 mM TBOOH are shown with the mean of three biological replicates represented with a colored line and the standard error represented as a shaded ribbon.

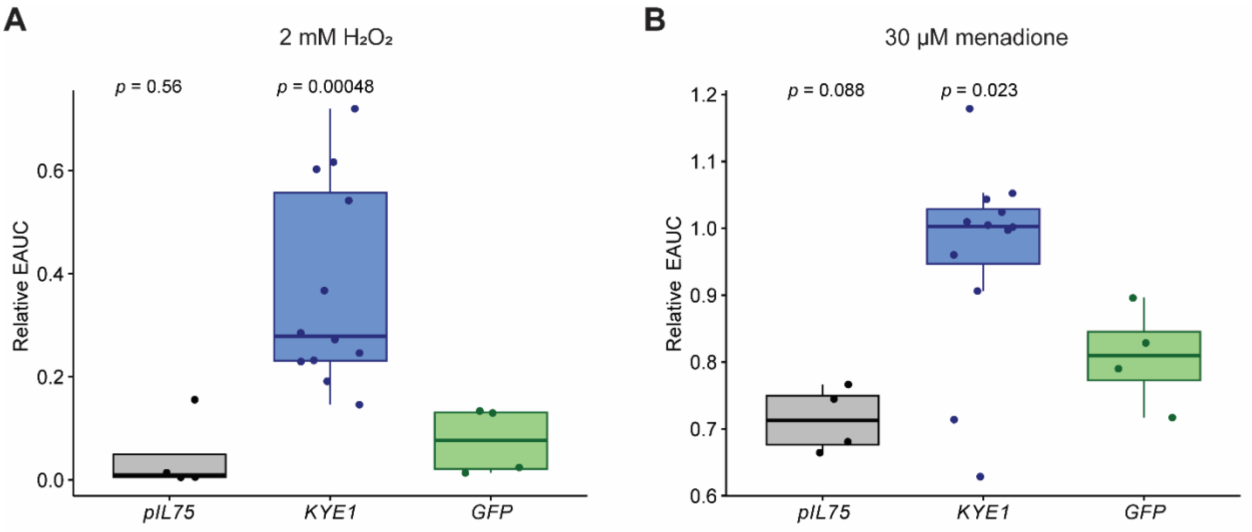

Supplementary Figure 5. *KYE1* contributes to resistance against multiple types of oxidative stress. *K. lactis* strains were grown in liquid SC+MSG+G418 medium with or without (A) 2 mM H_2O_2 or (B) 30 μM menadione in a 96-well plate format. The ROS resistance of these strains was compared using the EAUC in the oxidative stress agent relative to the untreated control. The points in the boxplots represent individual replicates (n=4 for *pIL75*, n=12 for *KYE1* and n=4 for *GFP*), and the boxes represent the interquartile ranges. The p-values are based on two-sided t-tests relative to the *GFP* control.

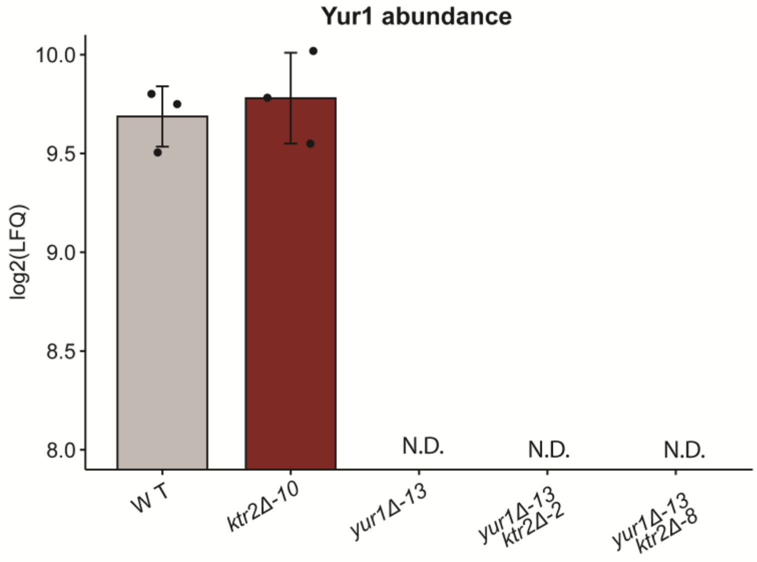

Supplementary Figure 6. The Yur1 protein is absent in deletion strains. There were no unique Yur1 peptides detected from the three strains of *S. cerevisiae* in which the *YUR1* gene was deleted. For the wild type (WT) and strain lacking *KTR2* only (*ktr2Δ-10*) the height of the bar represents the mean of the log_2-transformed label-free quantification (LFQ) of Yur1 for each strain, and the error bars represent the standard deviation. Each biological replicate (n=3) is shown as an individual dot, and N.D. stands for not detected.

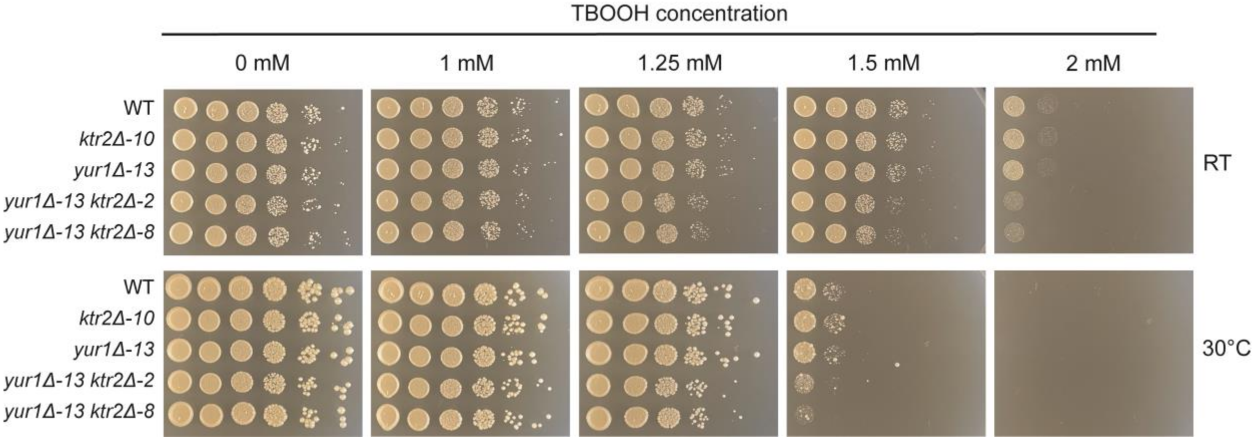

Supplementary Figure 7. Deletion of genes encoding mannosyltransferases (*MNT*) sensitizes *S. cerevisiae* to oxidative stress (A) A spot assay showing growth of the *S. cerevisiae MNT* deletion strains at varying concentrations of TBOOH as indicated (*KTR2* and *YUR1* are members of the *MNT* gene family). Plates were incubated at room temperature (RT or 22°C) or 30°C to reflect both our screening conditions and the normal laboratory growth conditions of *S. cerevisiae*, respectively. Plates were imaged after three days of growth and are representative of three replicates.

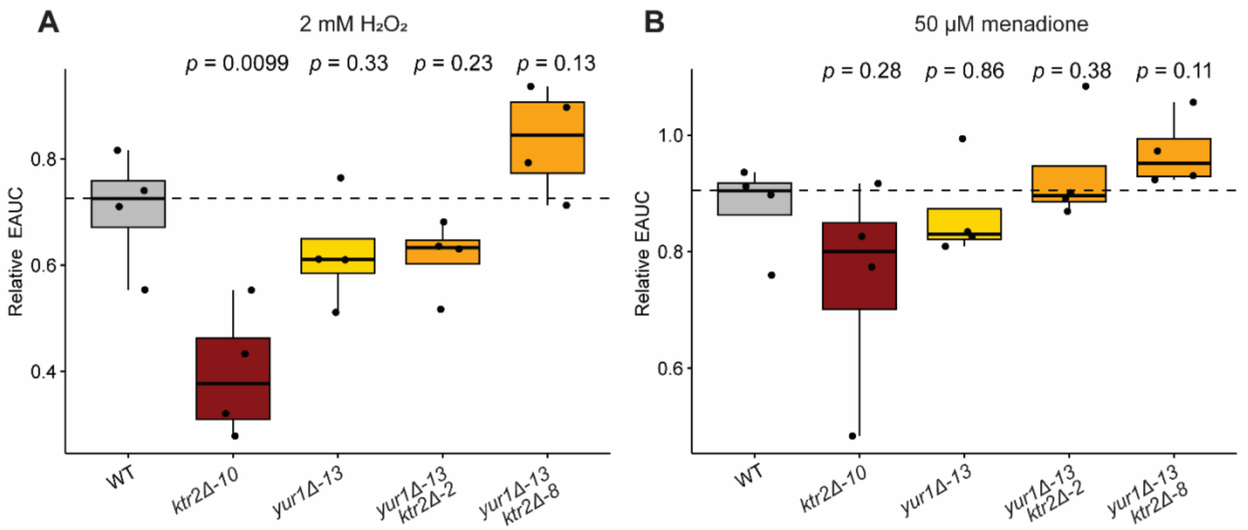

Supplementary Figure 8. Impact of mannosyltransferases on growth in multiple types of oxidative stress. *S. cerevisiae* strains were grown in liquid SC media with or without (A) 2 mM H_2O_2 or (B) 50 μM menadione in a 96-well plate format. The ROS resistance of these strains was compared using the EAUC in the oxidative stress agent relative to the untreated control. The points in the boxplots represent individual biological replicates (n=4), and the boxes represent the interquartile ranges. The p-values are based on two-sided t-tests relative to the WT control.

Supplementary Data

Supplementary Data 1. The species screened for their ROS resistance or sensitivity. The species' names, strain identifiers, taxonomic orders, and relative empirical area under the curve (EAUC) in both 1 mM and 2 mM concentrations of TBOOH are given.

Supplementary Data 2. The 50 top features identified by the machine learning model, including the orthologs of each gene family in *S. cerevisiae* and *C. albicans,* and the relative importance of each feature.

Supplementary Data 3. The SHAP values estimated for each of the 50 important features for each species included in the model.

Supplementary Data 4. The SHAP values estimated for the top 50 most predictive features for *S. cerevisiae*.

Supplementary Data 5. The input data matrix used for the machine learning model, including all species used in the model, their classifications, and the number of orthologs for each species in each orthogroup.

Supplementary Data 6. Primers used in this study.

Supplementary Data 7. Strains used and generated for validation experiments in this study.
