# Supplementary material for: Machine learning reveals genes impacting oxidative stress resistance across yeasts
Source: Nat Commun. 2025 Jul 1;16:5866. doi: 10.1038/s41467-025-60189-3 (PMC12215403; doi:10.1038/s41467-025-60189-3)
Supplement: Supplementary file 2 — Description of Addtional Supplementary Files [file 41467_2025_60189_MOESM2_ESM.pdf]

## Description of Additional Supplementary Files

**Supplementary Data 1.** The species screened for their ROS resistance or sensitivity. The species' names, strain identifiers, taxonomic orders, and relative empirical area under the curve (EAUC) in both 1 mM and 2 mM concentrations of TBOOH are given.

**Supplementary Data 2.** The 50 top features identified by the machine learning model, including the orthologs of each gene family in S. cerevisiae and C. albicans, and the relative importance of each feature.

**Supplementary Data 3**. The SHAP values estimated for each of the 50 important features for each species included in the model.

**Supplementary Data 4.** The SHAP values estimated for the top 50 most predictive features for S. cerevisiae.

**Supplementary Data 5.** The input data matrix used for the machine learning model, including all species used in the model, their classifications, and the number of orthologs for each species in each orthogroup.

**Supplementary Data 6.** Primers used in this study.

**Supplementary Data 7.** Strains used and generated for validation experiments in this study.
